# Supplementary material for: From sequence to enzyme mechanism using multi-label machine learning
Source: BMC Bioinformatics. 2014 May 19;15:150. doi: 10.1186/1471-2105-15-150 (PMC4229970; doi:10.1186/1471-2105-15-150)
Supplement: Additional file 2 — Java code of ml2db. Additional file ml2db_code.tar.gz contains the Java source code to run the multi-label machine learning experiments and save the results to database. The code’s Javadoc is included. [file 1471-2105-15-150-S2.zip › additional file 2/ml2db/ecmulan/doc/uk/ac/ed/inf/mulanxml/test/ec/EcNumberTest.html]

EcNumberTest


JavaScript is disabled on your browser.


- Overview
- Package
- Class
- Use
- Tree
- Deprecated
- Index
- Help

- Prev Class
- Next Class

- Frames
- No Frames

- All Classes

- Summary:
- Nested |
- Field |
- Constr |
- Method

- Detail:
- Field |
- Constr |
- Method


uk.ac.ed.inf.mulanxml.test.ec

## Class EcNumberTest

- java.lang.Object
- - junit.framework.Assert
  - - junit.framework.TestCase
    - - uk.ac.ed.inf.mulanxml.test.ec.EcNumberTest

- All Implemented Interfaces:
  :   junit.framework.Test

  ---

    

  ```
  public class EcNumberTest
  extends junit.framework.TestCase
  ```

  Class

  Version:
  :   30 Apr 2010

  Author:
  :   Luna De Ferrari luna.deferrari-at-ed.ac.uk

- - ### Constructor Summary

    Constructors

    Constructor and Description

    `EcNumberTest()`
  - ### Method Summary

    Methods

    | Modifier and Type | Method and Description |
    | --- | --- |
    | `static EcNumber` | `get0dashEcA()` |
    | `static EcNumber` | `get0dashEcB()` |
    | `static EcNumber` | `get1dashEc()` |
    | `static EcNumber` | `get2dashEc()` |
    | `static EcNumber` | `get3dashEc()` |
    | `static EcNumber` | `get4dashEc()` |
    | `void` | `testCompareTo()` |
    | `void` | `testGetBlock1()` |
    | `void` | `testGetBlock2()` |
    | `void` | `testGetBlock3()` |
    | `void` | `testGetBlock4()` |
    | `void` | `testGetHierarchy()` |
    | `void` | `testGetHierarchyLevel()` |
    | `void` | `testGetParent()` |
    | `void` | `testIsComplete()` |
    | `void` | `testIsParent()` |
    | `void` | `testSplitString()` |

    - ### Methods inherited from class junit.framework.TestCase

      `countTestCases, getName, run, run, runBare, setName, toString`
    - ### Methods inherited from class junit.framework.Assert

      `assertEquals, assertEquals, assertEquals, assertEquals, assertEquals, assertEquals, assertEquals, assertEquals, assertEquals, assertEquals, assertEquals, assertEquals, assertEquals, assertEquals, assertEquals, assertEquals, assertEquals, assertEquals, assertEquals, assertEquals, assertFalse, assertFalse, assertNotNull, assertNotNull, assertNotSame, assertNotSame, assertNull, assertNull, assertSame, assertSame, assertTrue, assertTrue, fail, fail, failNotEquals, failNotSame, failSame, format`
    - ### Methods inherited from class java.lang.Object

      `equals, getClass, hashCode, notify, notifyAll, wait, wait, wait`

- - ### Constructor Detail


    - #### EcNumberTest

      ```
      public EcNumberTest()
      ```
  - ### Method Detail


    - #### testCompareTo

      ```
      public void testCompareTo()
      ```


    - #### testGetBlock1

      ```
      public void testGetBlock1()
      ```


    - #### testGetBlock2

      ```
      public void testGetBlock2()
      ```


    - #### testGetBlock3

      ```
      public void testGetBlock3()
      ```


    - #### testGetBlock4

      ```
      public void testGetBlock4()
      ```


    - #### testGetHierarchy

      ```
      public void testGetHierarchy()
      ```


    - #### testGetHierarchyLevel

      ```
      public void testGetHierarchyLevel()
      ```


    - #### testGetParent

      ```
      public void testGetParent()
      ```


    - #### testIsComplete

      ```
      public void testIsComplete()
      ```


    - #### testIsParent

      ```
      public void testIsParent()
      ```


    - #### testSplitString

      ```
      public void testSplitString()
      ```


    - #### get0dashEcA

      ```
      public static EcNumber get0dashEcA()
      ```


    - #### get0dashEcB

      ```
      public static EcNumber get0dashEcB()
      ```


    - #### get1dashEc

      ```
      public static EcNumber get1dashEc()
      ```


    - #### get2dashEc

      ```
      public static EcNumber get2dashEc()
      ```


    - #### get3dashEc

      ```
      public static EcNumber get3dashEc()
      ```


    - #### get4dashEc

      ```
      public static EcNumber get4dashEc()
      ```


- Overview
- Package
- Class
- Use
- Tree
- Deprecated
- Index
- Help

- Prev Class
- Next Class

- Frames
- No Frames

- All Classes

- Summary:
- Nested |
- Field |
- Constr |
- Method

- Detail:
- Field |
- Constr |
- Method
